# Macrophages and angiogenesis in rheumatic diseases

**DOI:** 10.1186/2045-824X-5-11

**Published:** 2013-06-01

**Authors:** Nicola Maruotti, Tiziana Annese, Francesco Paolo Cantatore, Domenico Ribatti

**Affiliations:** 1 Rheumatology Clinic, Department of Medical and Surgical Sciences, University of Foggia Medical School- Ospedale “ D’Avanzo”, Foggia, Italy; 2 Department of Basic Medical Sciences, Neurosciences and Sensory Organs, University of Bari Medical School, Piazza Giulio Cesare, 11, Policlinico, 70124, Bari, Italy

**Keywords:** Angiogenesis, Arthritis, Connectivities, Macrophage, Vasculitides

## Abstract

Angiogenesis plays a key role in several rheumatic diseases, including rheumatoid arthritis, osteoarthritis, ankylosing spondylitis, systemic sclerosis, systemic lupus erythematosus, and vasculitides. An imbalance between angiogenic inducers and inhibitors seems to be a critical factor in pathogenesis of these diseases. Macrophages promote angiogenesis during rheumatoid arthritis. In addition, macrophages can produce a variety of pro-angiogenic factors that have been associated with the angiogenic response occurring during other rheumatic diseases. Lastly, macrophages could be a target in the treatment of rheumatoid arthritis and other rheumatic diseases. Nevertheless, further studies are needed to better elucidate the exact role of macrophage in angiogenesis in these diseases.

## Introduction

Macrophages are a population of cells derived from CD34 positive bone marrow progenitors, which differentiate to form blood pro-monocytes. Then pro-monocytes develop into monocytes and extravasate into tissues where they become “resident” tissue macrophages. Even if “resident” macrophages are characterized by different phenotypes within tissues, from that of Kupferr cells in the liver, microglial cells in the brain, and Langerhans cells in the skin, they share common aspects, such as their capacity to influence normal cell turnover and tissue remodeling, to counteract microbial infections, and to facilitate repair in sites of injury [1].

Macrophages may be recruited as consequence of any local disturbance of tissue homeostasis, including normal cell turnover or wounding, infections, immune response and malignancy. After recruitment, macrophages become “activated macrophages” showing different phenotypes in relation to the nature of the recruiting stimulus and the location.

As consequence of the variety of secretory products, anatomic diversity and functional heterogeneity, macrophages are involved in different physiological mechanisms and plays a key role in the aetiology and pathogenesis of numerous diseases. There are numerous evidences that macrophages are involved in both physiological and pathological angiogenesis [2,3].

Activated macrophages are generally categorized in two types, M1 (classically activated) and M2 (alternatively activated) [4,5]. M2 macrophages are further subdivided into M2a (activated by interleukin [IL]-4 or IL-13), M2b (activated by immune complexes in combination with IL-1β or lipopolysaccharide [LPS] and M2c (activated by IL-10, transforming growth factor-β [TGFβ] or glucocorticoids). M1 macrophages are able to kill microorganisms as well as tumor cells and secrete high levels of pro-inflammatory cytokines and tumoricidal agents, reactive nitrogen and oxygen intermediates , whereas the M2-derived chemokines play a role in the resolution of inflammation through phagocytosis of apoptotic neutrophils, reduced production of pro-inflammatory cytokines, and increased synthesis of mediators important in tissue remodeling, angiogenesis, and wound repair [4,5].

Considering the increasing interest for the role of angiogenesis in the pathogenesis of rheumatic diseases, such as rheumatoid arthritis, in this review we will focus on the role of macrophages in angiogenesis associated with rheumatic diseases.

### Angiogenesis

Angiogenesis is a process characterized by the formation of newly formed capillaries from pre-existing blood vessels. Angiogenesis is regulated by several angiogenic and antiangiogenic factors. About 30 angiogenic factors have been described, such as vascular endothelial growth factor (VEGF) family, fibroblast growth factor (FGF) family, TGF-α and -β), platelet-derived growth factor (PDGF), tumor necrosis factor alpha (TNF-α), angiogenin, Interleukins (ILs), chemokines and angiopoietins (Ang) [6,7]. On the other hand, several endogenous antiangiogenic factors have been identified, including angiostatin, endostatin, and thrombospondin (TSP). An imbalance between these positive and negative factors, with a prevalence of positive regulators, or a downregulation of the expression of negative regulators, is involved in pathological angiogenesis [6]. Physiological angiogenesis is characterized by a cascade of events which contains a number of distinct steps [8]. Angiogenic factors induce endothelial cell production of proteolytic enzymes, including matrix metalloproteinases (MMPs) and plasminogen activators, which are involved in the degradation of the basement membrane and of the perivascular extracellular matrix. Successively, endothelial cells proliferate and migrate into the perivascular area forming “primary sprouts”. The subsequent lumenation of these “primary sprouts” is responsible of the formation of “capillary loops”. At the same time, there is the synthesis of a new basement membrane, which is the final stage of new vessel formation. Successively, endothelial cells of the “primary sprouts” proliferate and migrate to generate secondary and further generations of sprouts.

In addition to this model, called “sprouting angiogenesis”, other models for angiogenesis have been described. The so-called not sprouting angiogenesis or intussusceptions is characterized by a column of interstitial cells which divides the lumen of a pre-existing vessel in two parts forming two vessels [9]. In tumors, other angiogenic mechanisms have been seen, such as vasculogenic mimicry and the formation of mosaic vessels. In vasculogenic mimicry, transdifferentiation of cancer cells allowing them to form tubular structures occurs. Mosaic vessels are characterized by the cooperation between endothelial cells and tumor cells to form new vessels [10,11].

Angiogenesis is involved in the pathogenesis of several diseases, including chronic inflammatory diseases. In fact, inflammatory infiltrates and newly-formed vessels have been described in chronic inflammatory diseases, including rheumatoid arthritis and vasculitides. In these pathological conditions, angiogenesis support inflammatory cells recruitment and determines a compensatory response to ischemia and to the augmented metabolic activity [12,13]. In fact, angiogenic agents promote endothelial cell expression of adhesion molecules and inflammatory cytokines and chemokines. VEGF may induce endothelial cells to express adhesion molecules, such as vascular cell adhesion molecule-1 (VCAM-1) and intercellular adhesion molecule-1 (ICAM-1), allowing the migration of monocytes and lymphocytes into the extracellular matrix [14,15]. Angiogenic factors, such as chemokines containing the ELR motif (glutamyl-leucyl-arginyl sequence), and CXC chemokines, are responsible for inflammatory cells recruitment [16,17]. Moreover, FGF-1 and FGF-2 favor migration of inflammatory cells via inducing endothelial cell to produce plasminogen activator and collagenase which are responsible for the degradation of the extracellular matrix [18].

It is interesting to note that most angiogenic agents, such as TNF-α, IL-1, IL-6, IL-8, and IL-18 are also inflammatory factors which are involved in increasing the production of other inflammatory cytokines and cell adhesion molecules, and in improving matrix cyclooxygenase activity and MMPs [19].

MMPs are secreted or membrane-anchored zinc-dependent endopeptidases, which are involved in the degradation of components of the extracellular matrix. Remodeling of the extracellular matrix by MMPs is important in angiogenesis. Among the various subtypes of MMPs, MMP-1, MMP-2 and MMP-9 expression levels have been demonstrated in rheumatoid synovial macrophages [20,21]. Moreover, synovial macrophages also express tissue inhibitors of MMPs (TIMPs) that contrast the effects of MMPs [20].

Members of the Wnt protein family have been shown to regulate several biological processes even if only recently its role in angiogenesis has been demonstrated [22,23]. By considering that both macrophages and secreted Wnt proteins regulate angiogenesis, it has been recently hypothesized that Wnt ligands mediate some of the effects that macrophages have on angiogenesis [2]. An augmented activation of Wnt5a has been seen in macrophages exposed to inflammatory agents, including interferon-γ (IFN-γ) and lipopolysaccharide (LPS) [2]. Moreover, Wnt5a may be responsible for inducing macrophage expression of numerous angiogenic cytokines, includingIL-6, IL-8 and IL-1β [20]. Wnt5a is also involved in inducing endothelial cell production of the monocyte chemoattractant protein-1 (MCP-1)/CCL2 [24].

On the other hand, there are evidences that Wnt proteins may mediate also antiangiogenic effects, probably because angiogenesis is characterized by different morphological changes required at different stage [2,25]. Thus, it is possible that the same protein may be angiogenic or antiangiogenic factor depending on the stage of angiogenesis at which it is present [2]. Furthermore, Wnt signal may be influenced by other pathways, such as VEGF, Ang-1 and ang-2, Notch/Dll4, and focal adhesion kinase (FAK) [26]. Moreover, differential Wnt receptor expression has been hypothesized in cells responding to the signal [2].

### The role of macrophages in angiogenesis in rheumatoid arthritis

Many angiogenic factors have been demonstrated in rheumatoid arthritis synovium, including CXC chemokines containing the ELR motif [27,28]. They bind to endothelial cells via specific endothelial chemokine receptors, such as CXCR2. In rheumatoid arthritis, CXCR2 has been demonstrated in synovial-tissue macrophages. CXCR2 is involved in monocyte recruitment from the circulation via binding to MCP-1/CCL2 [27,29,30]. Furthermore, CXCR2 recognizes important inflammatory chemokines, including growth-regulated oncogene a (groa)/CXCL1, IL-8/CXCL8, epithelial-neutrophil activating protein-78 (ENA-78)/CXCL5, connective tissue-activating peptide-III (CTAP-III)/CXCL7 and granulocyte chemotactic protein 2 (GCP-2)/CXCL6 [27,29,31]. Moreover, synovial-tissue macrophages express also CXCR4, the receptor of another important angiogenic chemokine, namely specific receptor for stromal cell-derived factor-1 (SDF-1)/CXCL12 [27,29].

Macrophage-derived angiogenic chemokines identified in rheumatoid arthritis synovial tissue include ENA-78/CXCL5 [32], a chemokine involved in the chemotaxis of neutrophils [33,34]. Fractalkine/CX3CL1, another chemokine produced by synovial macrophages, is involved in cell adhesion and in chemotaxis of monocytes and lymphocytes [27,29,35]. Groa/CXCL1, CTAP-III/CXCL7, and MCP-1/CCL2 [27,35,36], and macrophage inflammatory protein-1α (MIP-1α)/CCL3, responsible of apoptosis inhibition, increases macrophage and neutrophils infiltration, and induces angiogenesis in synovial tissue [37].

In rheumatoid arthritis synovium, macrophages may be activated by T cells via cell-cell contact. Moreover, numerous soluble mediators are involved in macrophage activation. In fact, immune complexes promote macrophage activation through the binding to FcγRs. Furthermore, peptidoglycans, LPS and heat-shock proteins may induce macrophage activation via binding to Toll-like receptors [38,39]. Activated macrophages are responsible for production of numerous angiogenic cytokines and growth factors. In rheumatoid arthritis synovial tissue, macrophages release VEGF through TNF-α, TGF-α, and IL-1 stimulation [40]. VEGF plays a key role in induction and amplification of angiogenic response [41]. VEGF receptors (VEGFRs) are expressed on macrophages, including constitutive expression of VEGFR-1, -3, and inducible expression of VEGFR-2. There are evidences that VEGFRs have a role in the recruitment of macrophages in various inflammatory conditions [41-46]. In rheumatoid arthritis, VEGFR-1 is responsible for macrophage activation and angiogenesis [27,28,47]. Moreover, VEGFR-1 deficiency results in decreased disease severity and diminished macrophage functions, such as phagocytosis and the secretion of IL-6 and VEGF-A, in murine models of arthritis [47]. Chung et al. [48] have hypothesized that VEGFR-3-specific signaling can induce new blood vessels, through the involvement of macrophages.

Other important macrophage-derived angiogenic factors are IL-15, IL-17, IL-18, TNF-α, FGF, PDGF, and IL-8 that enhances the expression of leukocyte adhesion molecule [27,28,33,49-51]. IL-15 and IL-18 are involved in T helper 1 polarization and, as recently demonstrated, IL-18 is also responsible for inducing macrophage production of MCP-1/CCL2 [52]. Among various PDGF isoforms, only PDGF-C and PDGF-D, and not PDGF-A and PDGF-B, are expressed by synovial fibroblasts and macrophages in rheumatoid arthritis synovial membrane. Moreover, PDGF-D induces synovial fibroblast proliferation and expression of MMP-1 [53]. Carmi et al. [54] have demonstrated that macrophage-derived IL-1β initiates angiogenesis by recruitment of cells of myeloid and endothelial lineages, especially in hypoxic condition.

In rheumatoid arthritis, hypoxia is induced by the high metabolic demands of synovial inflammation. An increased number of macrophages has been demonstrated in hypoxic tissues, such as synovial membrane, where hypoxia induces VEGF production by macrophages and other cells [27,28,55]. In fact, expression of hypoxia inducible factor-1α (HIF-1α) by macrophages has been found in rheumatoid synovial membrane, mostly close to the intimal layer and in the subintimal area [56]. The reduced intra-articular PO_2_ is responsible for inducing HIF-1α expression, which, in turn, induces synovial cells, macrophages, and other inflammatory cells to produce VEGF [57].

Moreover, macrophage migration-inhibitory factor (MIF) is expressed by macrophages in the synovium, where it is responsible for inducing macrophage production of angiogenic agents, including TNF-α, IL-1, IL-6, IL-8/CXCL8 and MMPs production [21,39]. In animal models of rheumatoid arthritis, MIF antagonism or deficiency result in decreased disease severity [58].

IL-6, LPS, IL-1β, IFN-α, IFN-γ and TNF-α induce CCAAT/enhancer-binding protein D (CEBPD) expression in rheumatoid arthritis [20,59-63]. CEBPD is a member of the family of the basic leucine zipper domain transcription factors, involved in tissue differentiation, metabolism and immune response. CEBPD activation in macrophage can promote angiogenesis [63], probably via activation of CEBPD-responsive factors, such as groa/CXCL1 and TNFAIP6 [64,65]. Groa/CXCL1 promotes microvascular endothelial cell tube formation *in vitro*[63,65]. TNFAIP6 contains a hyaluronan-binding LINK domain and a CUB (complement subcomponents C1r/C1s, Uegf, BMP-1) domain. The LINK domain, probably through inhibition of MMPs and aggrecanase enzymes, has shown chondroprotective effects in various models of inflammation and arthritis [63,64,66-68]. Thus, the angiogenic effect of TNFAIP6 is probably related to extracellular matrix remodeling to achieve regulation of vascular formation [63].

On the other hand, macrophages are involved in the production of important antiangiogenic factors, such as IP-10/CXCL10, Mig/CXCL9, IFN-γ, TIMPs, and TSP2. In rheumatoid arthritis, macrophages produce TSP2 in the lining layer and in the stroma of diffuse synovitis. On the contrary, macrophages do not produce TSP2 when they are organized in lymphoid microstructures. In fact, the less aggressive pattern of rheumatoid arthritis is characterized by diffuse synovitis and absence of organized lymphoid microstructures [69,70]. Moreover, macrophages may produce IL-27, a cytokine expressed in rheumatoid arthritis synovium [70,71]. Using a murine model of collagen-induced arthritis (CIA), Pickens et al. [72] have demonstrated that IL-27 expression results in reduced synovial vasculature, probably due to downregulation of IL-17 levels in joints with forced IL-27 expression. IL-27 over-expression is responsible for inhibiting IL-1β and IL-6 production, and this leads to a reduced T helper-17 activity characterized by decreased IL-17 expression. Low levels of IL-17 are responsible for a decreased synovial production of macrophage-derived angiogenic factors, such as groa/CXCL1, ENA-78/CXCL5, and MCP-1/CCL2.

### The role of macrophages in angiogenesis in other rheumatic diseases

Studies on the role of macrophages in angiogenesis in other rheumatic diseases are very scarce. In osteoarthritis, angiogenesis enhances inflammation and contributes to the severity of the disease. Angiogenesis may be found in osteochondral junction and synovium from patients with osteoarthritis, where macrophages may contribute to angiogenesis via VEGF expression [73,74]. A role of VEGF has been described in psoriatic arthritis and ankylosing spondylitis [75]. In systemic sclerosis, VEGF has been detected in sera of patients, even if the role of angiogenesis is controversial [75,76]. VEGF, epidermal growth factor (EGF), FGF and IL-18 have been found in sera of patients affected by systemic lupus erythematosus [75,77].

Several studies have demonstrated that angiogenesis plays a key role in the pathogenesis of vasculitides, such as giant cell arteritis, thromboangiitis obliterans, Kawasaki syndrome, Churg-Strauss syndrome, Wegener granulomatosis, microscopic polyangiitis, and Behcet disease. The angiogenic response is more evident in small vessel vasculitides than in medium- and large-vessels vasculitides, since angiogenesis generally involves capillary and post-capillary venules.

In vasculitides, angiogenesis may represent a compensatory response to ischemia and to increased metabolic activity principally in acute phase of disease [78]. A role for VEGF, FGF-2, TGF-β, PDGF, TNF-α, MCP-1, IL-6 and IL-8 have been described in giant cell arteritis [79,80]. Multinucleated giant cells (MGCs) are specialized fused cells derived by macrophages, which have been found in media-intima of arterial walls from patients affected by giant cell arteritis [81]. MGCs produce numerous angiogenic factors, such as VEGF and PDGF, and may be also involved in elastic membranes degradation via MMP-2 production [82-85].

In thromboangiitis obliterans, increased levels of TNF-α have been found in vascular lesions [86]. VEGF and TGF-β overexpression has been found in Kawasaki syndrome [75], and TGF-β1 upregulates VEGF expression in acute phase of disease [87].

Increased levels of TGF-β are also been detected in sera of patients affected by ANCA associated vasculitides, such as Churg-Strauss syndrome, Wegener granulomatosis, and microscopic polyangiitis [75,77].

In Behcet disease, increased IL-8 expression has been found in synovial fluids. Moreover, increased VEGF levels has been demonstrated in oral aphthous lesions, in the ocular inflammation and in blood serum [88-91].

### Macrophage: a therapeutic target in rheumatic diseases

Macrophage-derived chemokine production and pathological angiogenesis in rheumatic diseases may be suppressed by several antirheumatic drugs, including methotrexate, sulfasalazine, leflunomide, chloroquine, and anti-TNF agents [27-29]. These compounds may inhibit synovial vessel formation by nonspecifically blocking the action of angiogenic mediators [27,75]. A more favorable response after intra-articular glucocorticoid therapy or radiation synovectomy has been described in synovial membrane characterized by elevated synovial macrophage number [92]. Inhibition of TNF-α, IL-1β and IL-6 has been described in LPS stimulated human monocyte/macrophage after incubation with chloroquine [93]. Infliximab, a chimeric monoclonal antibody directed against TNF-α, in combination with methotrexate, leads to decreased synovial and skin VEGF expression in patients affected by psoriatic arthritis [94]. Moreover, the anti-IL-6 receptor antibody tocilizumab may reduce VEGF production in rheumatoid arthritis [95]. Thalidomide, recently introduced into the treatment of rheumatoid arthritis and lupus, is responsible for angiogenesis and TNF-α inhibition [27,75].

Moreover, improvement or resolution of arthritis in murine models has been seen after treatment with antibodies to macrophage-derived angiogenic chemokines, including IL-8, ENA-78/CXCL5, MIP-1α/CCL3, MCP-1/CCL2, and fractalkine [35,96,97]. Several oral chemokine receptor antagonists, including CXCR2 and CXCR4 inhibitors, have been tried in human rheumatoid arthritis as well as in animal models of arthritis [98]. Moreover, numerous chemokine receptor antagonists, including the nonpeptide antagonist of the murine CCR1, called J-113863, have reduced articular inflammation in murine collagen-induced arthritis, together to a reduction of TNF-α production by macrophages [99]. Encouraging results have been also seen in humans treated with a CCR1 antagonist in a phase Ib clinical trial [100]. Imatinib mesylate, a competitive tyrosine-kinase inhibitor used in the treatment of numerous hematological malignancies, seems to inhibit macrophage activation, osteoclastogenesis and joint damage in murine models of collagen-induced arthritis [101,102]. Dehydroxymethylepoxyquinomicin, a newly developed compound that inhibits nuclear factor κB activation, may inhibit macrophage cytokine production and suppress murine collagen-induced arthritis [103].

HIF-mediated angiogenesis may be a further target. YC-1, a superoxidesensitive stimulator of soluble guanylyl cyclase initially used as vasodilator in hypertension and thrombosis treatment, also diminishes HIF-1α expression and activity [104,105]. Paclitaxel, a mitotic inhibitor used in cancer chemotherapy, is also a HIF-1 inhibitor which has been proposed in rheumatoid arthritis treatment [104,106].

## Conclusions

**Figure 1:**
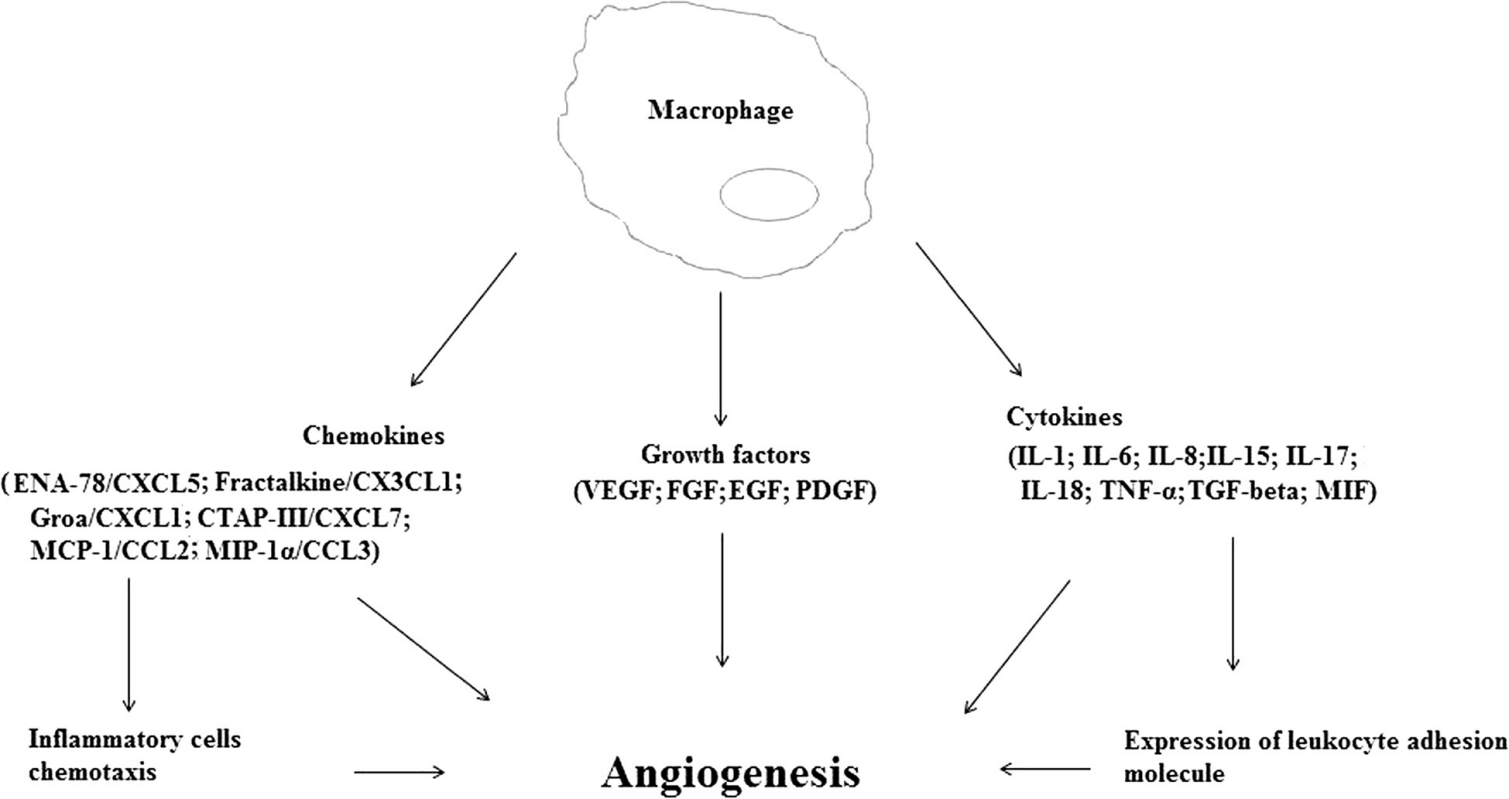
Chemokines, growth factors and cytokines involved in the angiogenic activity of macrophages.

Angiogenesis is emerging key player in pathogenesis of several rheumatic diseases, such as rheumatoid arthritis, osteoarthritis, ankylosing spondylitis, systemic sclerosis, systemic lupus erythematosus, and vasculitides. Among major cell types involved in angiogenesis, macrophages are known to produce numerous angiogenic factors, including VEGF, FGF, TGF-β, PDGF, TNF-α, MCP-1, IL-6, IL-8, and IL-18 (Figure 1).

Much research has been concentrated on the role of macrophage derived angiogenic factors in rheumatoid arthritis. Nevertheless, it is conceivable that macrophage may be involved in angiogenesis in other rheumatic diseases characterized by the presence of angiogenic factors which may be produced by macrophage, although not exclusively.

This suggests that macrophage could be usefully selected as a therapeutic targets of an antiangiogenic therapy in the treatment of rheumatic diseases, even if further studies are needed to better elucidate the exact role of macrophage in angiogenesis in these diseases.

## Abbreviations

ANCA: Antineutrophil cytoplasmic antibodies; CEBPD: CCAAT/enhancer-binding protein D; CIA: Collagen-induced arthritis; CTAP-III: Connective tissue-activating peptide-III; CUB: Complement subcomponents C1r/C1s, Uegf, BMP-1; ENA-78: Epithelial-neutrophil activating protein-78; FGF: Fibroblast growth factor; FAK: Focal adhesion kinase; GCP-2: Granulocyte chemotactic protein 2; HIF-1α: Hypoxia inducible factor-1α; IL: Interleukin; IFN-γ: Interferon-γ; MIF: Macrophage migration-inhibitory factor; MIP-1α: Macrophage inflammatory protein-1α; MMPs: Metalloproteinases; MCP-1: Monocyte chemoattractant protein-1; MGCs: Multinucleated giant cells; PDGF: Platelet-derived growth factor; SDF-1: Stromal cell-derived factor-1; TSP2: Thrombospondin 2; TIMPs: Tissue inhibitors of metalloproteinases; TGF: Transforming growth factor; TNF-α: Tumor necrosis factor alpha; VEGF: Vascular endothelial growth factor; VEGFRs: VEGF receptors.

## Competing interests

The authors declare that they have no competing interests.

## Authors’ contribution

NM and DR designed the study and write the paper. TA and FPC revised the manuscript. All authors read and approved the final manuscript.
